# Association between autonomic function, metabolic syndrome, and cognitive function in cognitively normal older adults in U.S. POINTER

**DOI:** 10.1002/alz70860_103400

**Published:** 2025-12-23

**Authors:** Hossam A Shaltout, Katelyn R Garcia, Laura D Baker, Heather M Snyder, Kathryn V Papp, Margie J Bailey, Tina E Brinkley

**Affiliations:** ^1^ Wake Forest University School of Medicine, Winston‐Salem, NC, USA; ^2^ Alzheimer's Association, Chicago, IL, USA; ^3^ Massachusetts General Hospital, Harvard Medical School, Department of Neurology, Boston, MA, USA

## Abstract

**Background:**

Metabolic syndrome (MetS), a cluster of interrelated risk factors including central obesity, hyperglycemia, dyslipidemia and hypertension, is increasingly recognized for its impact on cardiovascular health and cognitive function. However, the degree to which MetS associates with autonomic function and modifies risk for Alzheimer's disease (AD) in older adults is unknown. We investigated the association between MetS, cognition, and autonomic function in 440 participants enrolled in the U.S. POINTER trial (mean age: 68.1±5.3 years, 61% female, 26% people of color).

**Method:**

MetS was defined according to Adult Treatment Panel III (ATP III) criteria. Continuous blood pressure and ECG recordings were measured in the standing position and used to calculate baroreflex sensitivity (BRS), blood pressure variability (BPV), and heart rate variability (HRV) assessed as root of mean square of successive differences (rMSSD) and standard deviation of beat to beat interval (SDNN). A cognitive battery was administered to assess global and domain‐specific cognitive function. Autonomic and cognitive outcomes were compared by MetS status. Linear regression was used to examine cross‐sectional associations between the outcomes of interest adjusting for age, sex, race, ethnicity, education, and site.

**Result:**

Participants with MetS (*n* = 281, 63.8%) were younger (*p* = 0.009), likely to be male (*p* <0.0001), and had lower global cognition (*p* = 0.0018), episodic memory (*p* = 0.011), executive function (*p* = 0.005), and processing speed (*p* = 0.033). Autonomic function was reduced in MetS as measured by lower BRS (*p* = 0.034), lower SDNN (*p* = 0.01), higher BPV (*p* = 0.024), and a trend for lower rMSSD (*p* = 0.067). Similarly, in linear regression analyses, MetS was negatively associated with BRS (*p* = 0.007), rMSSD (*p* = 0.044), and SDNN (*p* = 0.0007) and positively associated with BPV (*p* = 0.009). Both rMSSD and SDNN were positively associated with global cognition (*p* = 0.039, *p* = 0.001) and executive function (*p* = 0.038, *p* = 0.002). SDNN was also positively associated with episodic memory (*p* = 0.004). Associations between autonomic and cognitive function were similar in participants with and without MetS.

**Conclusion:**

In this population of older adults at risk for AD, measures of healthy autonomic function are positively associated with cognition and negatively associated with MetS. Further studies are needed to assess if early diagnosis and treatment of MetS in this population could delay the cognitive decline.

